# Convulsive Tic

**Published:** 1905-03-25

**Authors:** 


### CONVULSIVE TIC.

Convulsive tic or habit-spasm is always a distressing malady, and occasionally becomes sufficiently intolerable to prompt self-destruction, as in one or two instances quoted by Dr. H. T. Patrick.[1] This author observes that habit-spasm is differentiated from other spasmodic movements by the fact that the repeated movement is always the same (though in course of time adjoining groups of muscles may be drawn into the spasmodic movement), and by the fact that the action is under partial control. He believes that the affection is sensory in origin, and that in children at least this sensory origin can often be traced. For instance, a child has an attack of mild conjunctivitis, which tempts him to blink repeatedly for some days; the blinking gives him momentary gratification, a gratification he may seek to prolong after all trace of the local lesion has vanished. In the same way a tight coat may originate a spasm of the shoulder, or a rubbing collar a spasmodic torticollis.

Dr. Patrick considers that the affection always indicates a neuropathic tendency, for, as he truly says, if a conjunctivitis, or an ill-fitting collar, or a coat tight at the shoulder, might be relied upon to produce a tic, the civilised world would be a remarkably jerky community. A curious point about the affection is that no matter how irresistible may be the impulse, the gratification of it is always sufficiently controlled to prevent derangement of voluntary movements: thus, a writer or a painter so affected, when he feels the impulse approaching, will lay down his pen or brush, gratify himself with a tic or two and resume his labours. This, of course, is a state of things far removed from the expression of chorea, or of true involuntary spasm.

Of the prognosis he says that in children it is good; in adults, bad. The treatment in the case of young patients resolves itself into removal of any discoverable source of irritation, with the employment of a judicious scheme of reward and penalty. The *rationale* of this method is to cultivate by constant reminders a corrective impulse which may be automatically called into operation by the advent of the impulse towards spasm. In this way each call to spasm successfully resisted reduces the force of the succeeding impulse in the same direction, until the morbid inclination dies of inanition, as one might say. We may observe that some physicians have achieved a measure of success in the treatment of this condition in young patients by means of alkalies, as happened in the following case:—A sharp, intelligent boy of 12 years acquired a spasm consisting of a forcible closure of one eye—blepharospasm. He was treated with bromides for a week without result. His conjunctiva was then desensitised with cocaine, because he attributed his blinking to a sense of irritation in the eye. This was not even temporarily successful. He was then sent to bed for a week, his eye being constantly kept closed by strapping, but at the expiry of that period not only was he no better, but the corner of his mouth and also the opposite eye were becoming involved in the movement. He was now given a mixture containing bicarbonate of soda and citrate of potash, and in less than a week all trace of spasm had disappeared. However little conclusive, this instance is suggestive enough to make the alkali treatment well worthy of trial in a recalcitrant case.

In adults Dr. Patrick believes that operative interference, such as is practised for spasmodic torticollis, is foredoomed to failure. No method of treatment is consistently satisfactory, though now and then hopes are raised by an isolated success along certain particular lines. The two systems upon which he lays most weight are, firstly, the keeping of the patient almost constantly asleep for several weeks at a stretch, by alternating administration of veronal, trional, chloral, and bromides (after Dr. Bastian); and, secondly, a system of graduated exercises to restore control of the affected muscles (Meige and Feindel).

[1] Jour. of Amer. Med. Assoc, Feb. 11, 1905.

